# Developing a pyroptosis-related gene signature to better predict the prognosis and immune status of patients with head and neck squamous cell carcinoma

**DOI:** 10.3389/fgene.2022.988606

**Published:** 2023-01-04

**Authors:** Dan Liu, Liu-Qing Zhou, Qing Cheng, Jun Wang, Wei-Jia Kong, Su-Lin Zhang

**Affiliations:** ^1^ Department of Otorhinolaryngology, Union Hospital, Tongji Medical College, Huazhong University of Science and Technology, Wuhan, China; ^2^ Institute of Otorhinolaryngology, Union Hospital, Tongji Medical College, Huazhong University of Science and Technology, Wuhan, China

**Keywords:** pyroptosis, HNSCC, prognosis, TCGA, immune microenvironment

## Abstract

Chronic inflammation may promote the incidence and development of neoplasms. As a pro-inflammatory death pathway, pyroptosis could induce normal cells to transform into cancerous cells, but the potential effect of pyroptosis in head and neck squamous cell carcinoma (HNSCC) remains unclear. This study developed and evaluated a pyroptosis-related gene signature to predict the prognosis and immune status of patients with HNSCC. The gene expression, mutation information, and clinical characteristics of HNSCC were extracted from TCGA to establish a comprehensive genome database (GEO). Based on LASSO Cox regression model, nine pyroptosis-related genes (TTLL1, TRIML2, DYNC1I1, KLHL35, CAMK2N1, TNFRSF18, GLDC, SPINK5, and DKK1) were used to construct a pyroptosis-related gene signature, which had good ability to predict the prognosis of HNSCC. Furthermore, the expression of nine pyroptosis-related genes in HNSCC and paracancerous tissues was detected by quantitative real-time PCR (qRT-PCR). The potential immunotherapeutic features and drug sensitivity prediction of this signature were also explored. Because pyroptosis regulators play an important role in HNSCC development and prognoses, further exploration might assist in identifying new biomarkers and predictors of prognosis to benefit clinical identification and management.

## Introduction

Head and neck squamous cell carcinoma (HNSCC) is ranked the sixth most common cancer globally (Ferlay et al., 2019; Siegel et al., 2020). It accounts for about 3% of new cancer cases (Siegel et al., 2014) and is generally related to tobacco and alcohol consumption (Johnson et al., 2020; von Witzleben et al., 2020). HNSCC is the most common malignant tumor in the head and neck and develops in the mucosal epithelium of the oral cavity, pharynx, and larynx (Hashibe et al., 2007; Raj et al., 2022). Despite the huge progress in the screening, diagnosis, surgery, chemotherapy, radiotherapy, immunotherapy, and molecular-targeted agents, the clinical outcomes of patients with HNSCC are unsatisfactory, with most patients not being cured (Leemans et al., 2011). This poor prognosis is related to native invasion, neck lymph node metastases, and local recurrence (Warnakulasuriya, 2009; Shah et al., 2010). Currently, the TNM Classification of Malignant Tumors (TNM) stage of disease is the crucial prognostic factor for the overall survival of HNSCC patients (Specenier and Vermorken, 2018). However, the survival rate of HNSCC patients has not significantly improved, emphasizing the need for reliable predictive biomarkers (Biomarkers Definitions Working Group, 2001) and new treatment strategies (Takes et al., 2012; Van den Bossche et al., 2022). In addition, several studies have revealed that gene mutations and molecular pathological subtypes considerably impact the prognosis of HNSCC patients. Consequently, it is essential to identify innovative prognostic markers and treatment targets to improve the low survival rate of HNSCC patients.

Pyroptosis, referred to as cell inflammatory necrosis, is a procedural cell death (Frank and Vince, 2019). It mainly mediates inflammasome-activated caspases to act with caspase-1, causing continuous cell extension and rupture, leading to the release of cellular substances and eventually a strong inflammatory response (Rühl et al., 2018; Kist, 2021; Yu et al., 2021). Pyroptosis is related to numerous diseases and is considered a “double-edged sword” in cancers (Kolb et al., 2014; Wang et al., 2019a; Wang et al., 2021). Although the pyroptosis inflammatory response environment may accelerate tumor growth in different cancers, it may also promote tumor cell apoptosis (Wang et al., 2019b; Wu et al., 2021). Recently, the role of pyroptosis in tumors has received widespread attention, with pyroptosis now considered to promote tumor immunotherapy effects (Wang et al., 2020; Tan et al., 2021a; Tan et al., 2021b). In particular, the large number of bacteria and viruses in the oral cavity, pharynx, and larynx can increase the chance of infection, along with the occurrence of pyroptosis. Although pyroptosis-related genes (PRGs) have prognostic value in predicting the outcomes of HNSCC patients (Shen et al., 2021; Zhu et al., 2022), PRGs-mediated immune infiltration and drug sensitivity are unclear.

This study aimed to investigate the expression of apoptosis-related genes, prognosis, association with immune status, and the diversity of responses to immunotherapy in HNSCC.

## Materials and methods

### Date collection

Gene expression and clinical features of HNSCC samples were collected from the publicly available Cancer Genome Atlas (TCGA) and Gene Expression Omnibus (GEO, https://www.ncbi.nlm.nih.gov/geo/).

### Patients and samples

A total of 413 patients with HNSCC were enrolled, and the collected clinicopathological data included the patient’s age, gender, stage, survival, and TNM classification. Of 413 patients, 246 were alive, 167 were dead, 291 were aged ≤65 years, 121 were aged >65 years, and one was unknown. There were 101 females and 312 males, 285 patients were in grades I and II, 109 patients were in grades III and IV, and 19 patients were unknown. In total, 72 patients had stages I and II, 274 had stages III and IV, and 67 were unknown. There were 185 N0 and N1 patients, 152 N2 and N3 patients, and 76 patients were unknown, with 148 T0, T1, and T2 cases, 210 T3 and T4 cases, and 55 patients were unknown. There were 151 M0 patients, one M1 patient, and 261 patients were unknown. Differentially expressed PRGs in normal and HNSCC tissues were determined using R software and the Wilcoxon test.

### Construction and validation of the prognostic pyroptosis-related gene signature

First, the differentially expressed genes (DEGs) in normal and HNSCC tissues were identified, then gene ontology (GO) and Kyoto encyclopedia of genes and genomes (KEGG) analyses were conducted. Cox univariate analysis and LASSO Cox regression were then applied to identify the prognostic-related genes using “glnet” R package (*p* < 0.05). Finally, nine PRGs were identified to construct the signature for prognostic PRG model development. The risk score of each patient was calculated based on the gene expression level associated with pyroptosis and the regression coefficient. HNSCC patients in TCGA cohort were then classified as low- and high-risk groups based on their median risk score. R Survminer package for survival analysis was used to determine the different OS, and the survival and time ROC R package was used to assess the predictive accuracy. Correlation analysis of immune cells in different software and drug sensitivity differences in high and low-risk groups was also performed.

### Expression of pyroptosis-related genes in tissues by qRTPCR

A total of 20 matched HNSCC and paracancerous tissues were obtained from Wuhan Union Hospital. Pathologists histopathologically confirmed the diagnosis of all tissues. All patients had not received chemotherapy, radiotherapy, targeted drugs, immunotherapy, or Chinese herbal medicine. Patients were not diagnosed with malignancy at other sites or with other serious underlying diseases. The Ethics Committee of Wuhan Union Hospital authorised this research (No: 20220076). All patients signed the informed consent form before surgery. The specimens were removed and rapidly frozen in liquid nitrogen and stored in a low-temperature refrigerator at −80°C for subsequent studies. Total RNA was extracted with a RNeasy mini kit (Axygen, United States) according to the manufacturer’s instructions. cDNA was reverse transcribed by a PrimeScript RT reagent kit with gDNA Eraser (TaKaRa, Japan, Code No. RR047A). The RNA and cDNA of each sample were analyzed by a GeneQuant pro RNA Calculator to assess the concentrations and purity. Quantitative real-time PCR was performed with real-time SYBR Green PCR reagents (Q311-02, Vazyme, Nanjing, China) and the 7300 Real-Time PCR System (Applied Biosystems, Foster City, CA). The abundance of different transcripts was assessed in triplicates.

### Statistical analysis

The data were analyzed using R (version 4.0.5) by Bioconductor packages. Derivation of prognostic signatures compared to different clinicopathological features of HNSCC was accessed using ROC curve analysis (Heagerty et al., 2000). The independent prognostic value of OS clinical features was assessed by Cox proportional risk regression analyses, and Kaplan-Meier was used to evaluate the survival analysis of HNSCC patients. The “limma R” package was used for differential analysis, whereas the “ConsensusCluster-Plus R”, “CIBERSORT”, and “ESTIMATE” R were used to analyze immune infiltration. The prediction model was constructed, applied, and validated using “timeROC R”, “survival R”, and “glmnet R” packages in HNSCC. In qRTPCR verification experimental, the relative expression was calculated based on the comparative Ct (2^−ΔΔCT^) method, and Student’s t-test (two-tailed) was utilized to assess the significance of gene expression differences in GraphPad Prism (version 8.0).

## Results

### Genetic variation prognoses of pyroptosis regulators in HNSCC

**FIGURE 1:**
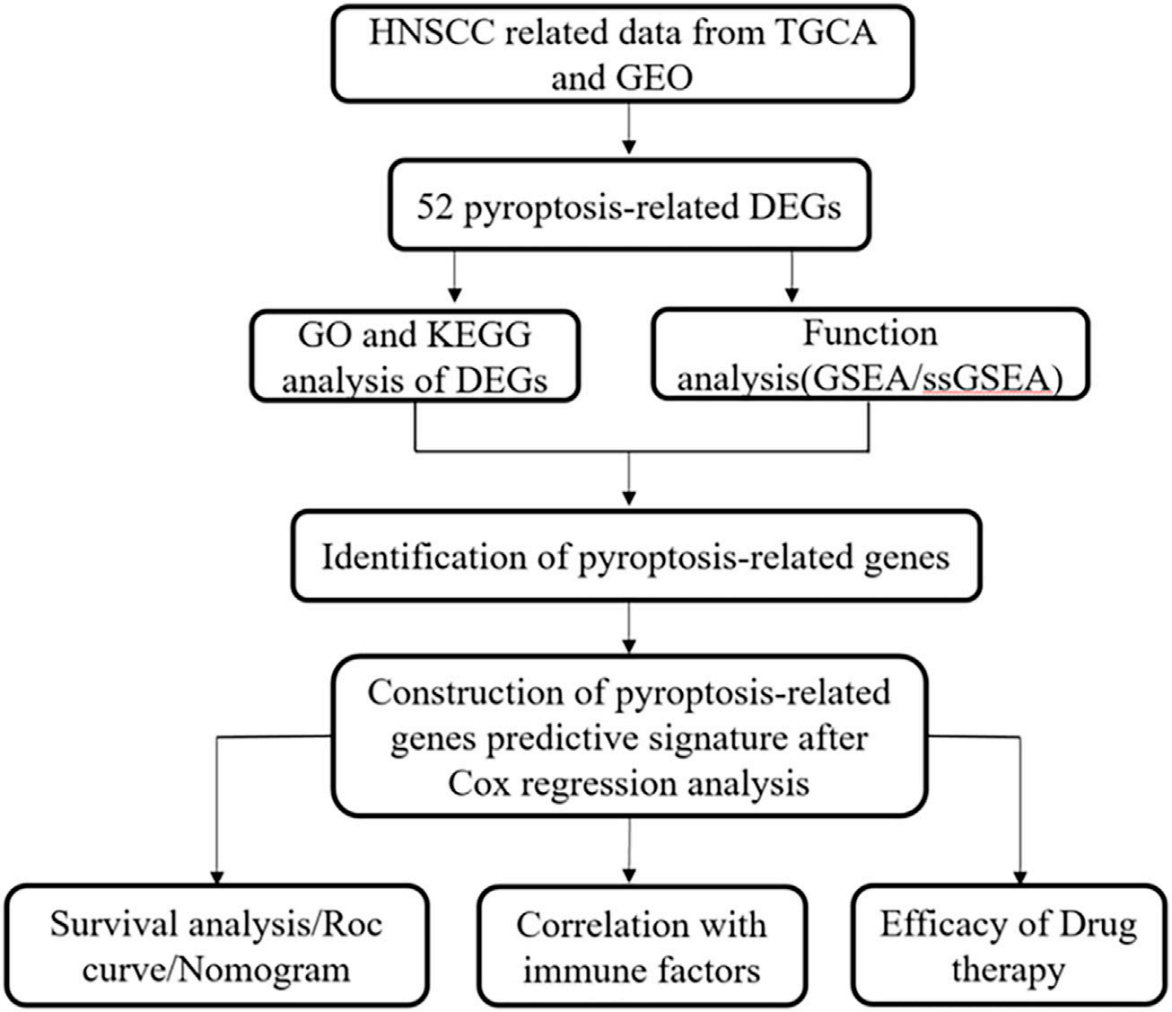
Study flow chart and scheme.

**FIGURE 2:**
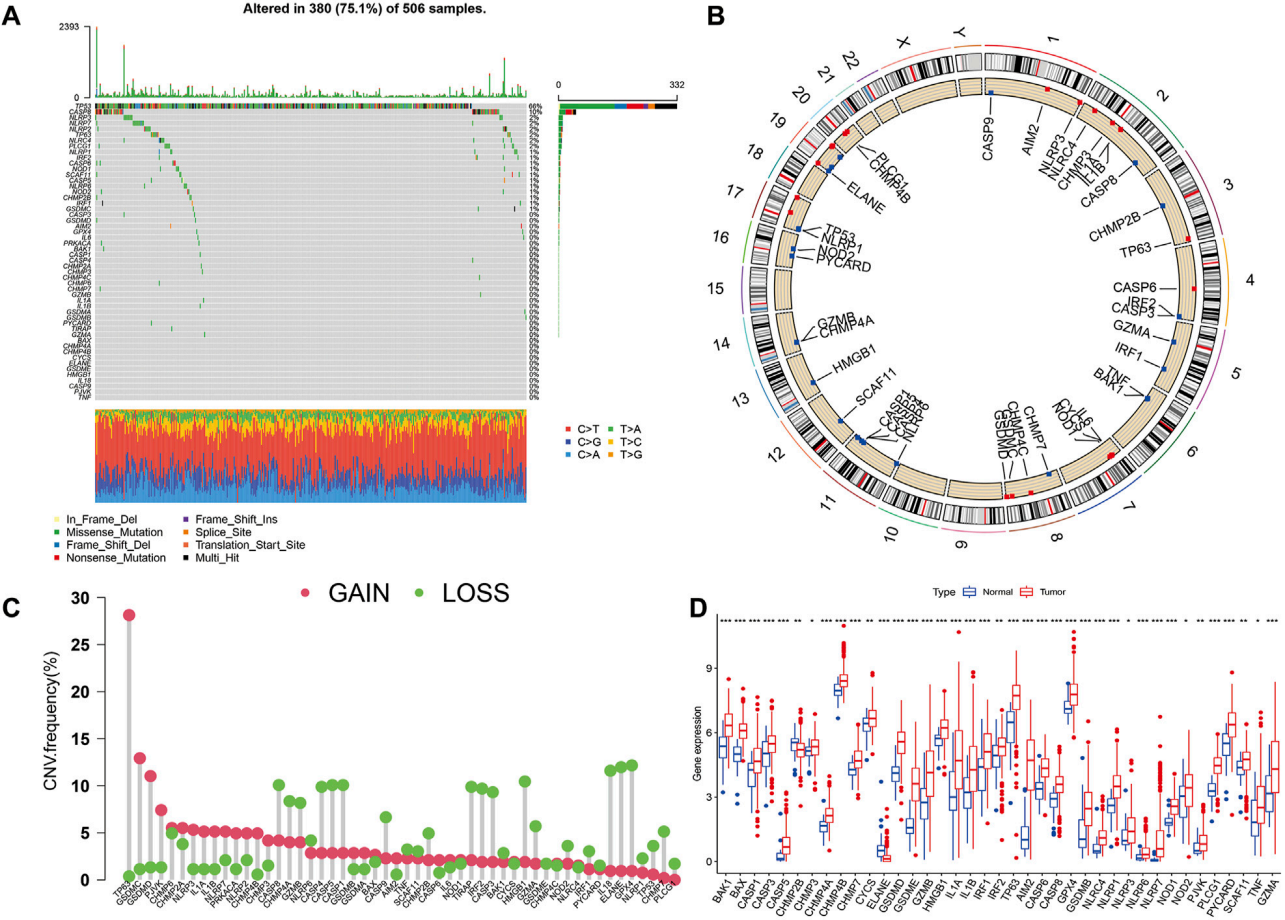
Expression and variation of pyroptosis regulators in HNSCC. **(A)** Mutation frequency of 52 PRGs for 506 HNSCC patients. **(B)** The location of PRGs on chromosomes in TCGA cohort. **(C)** Frequency of CNV alteration in PRGs. Green: missing frequency; red: amplified frequency. **(D)** The expressions of 52 pyroptosis regulatory factors differ between HNSCC and normal tissues. The upper and lower ends of the boxes represent the interquartile range of values. The lines in the boxes represent the median value. Tumor, red; Normal, blue. Statistically significant values are represented by asterisks corresponding to *p < 0.05, **p < 0.01, ***p < 0.001 and ****p < 0.0001.

**FIGURE 3:**
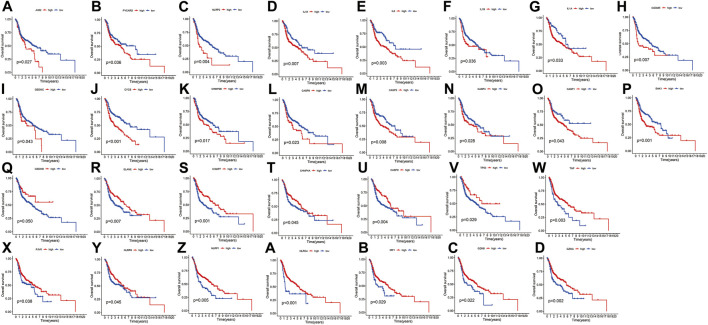
Kaplan-Meier curve for OS of 30 pyroptosis regulators. **(A–Z, A–D)** The association between 16 pyroptosis regulators with positive regulation and 14 with negative regulation and survival.

Figure 1 presents a flow chart of this study scheme. Fifty-two PRGs were identified after merging GEO and TCGA databases. Of 506 samples, 380 had a mutation rate of 75.1%. It was found that only BAX, CHMP4A, CHMP4B, CYCS, ELANE, GSDME, HMGB1, IL18, CASP9, PJVK, and TNF had no mutations, and others showed mutations in HNSCC tissues (Figure 2A). Figure 2B demonstrates copy number variation (CVN) alterations on PRG chromosomes. Additionally, CNV changes were common in 39 genes, less focused on increasing copy number, and TP63 gain was the most significant. Furthermore, the number of copies increased by two-thirds (Figure 2C). To determine whether these genetic variations affect PRG expression in HNSCC, PRG expression was further analyzed in tumor and normal tissues, indicating that the expression of 39 genes was significantly different between normal and tumor specimens (Figure 2D). PRG expression deleted in CNV was higher in cancer samples than in normal tissues (Figures 2C, D). The previous results revealed that although PRG expressions in tumors and normal tissues are highly heterogeneous, CNV change may not be the dominant factor leading to PRG disturbance. Survival analysis was performed to determine the impact of these 52 PRRs on HNSCC patients’ prognosis, implying that 30 PRGs were significantly different, 16 PRGs were negatively related to prognosis, and 14 pyroptosis regulators positively linked to survival (Figure 3).

### Identification of the immune features and prognoses of each pyroptosis cluster

**FIGURE 4:**
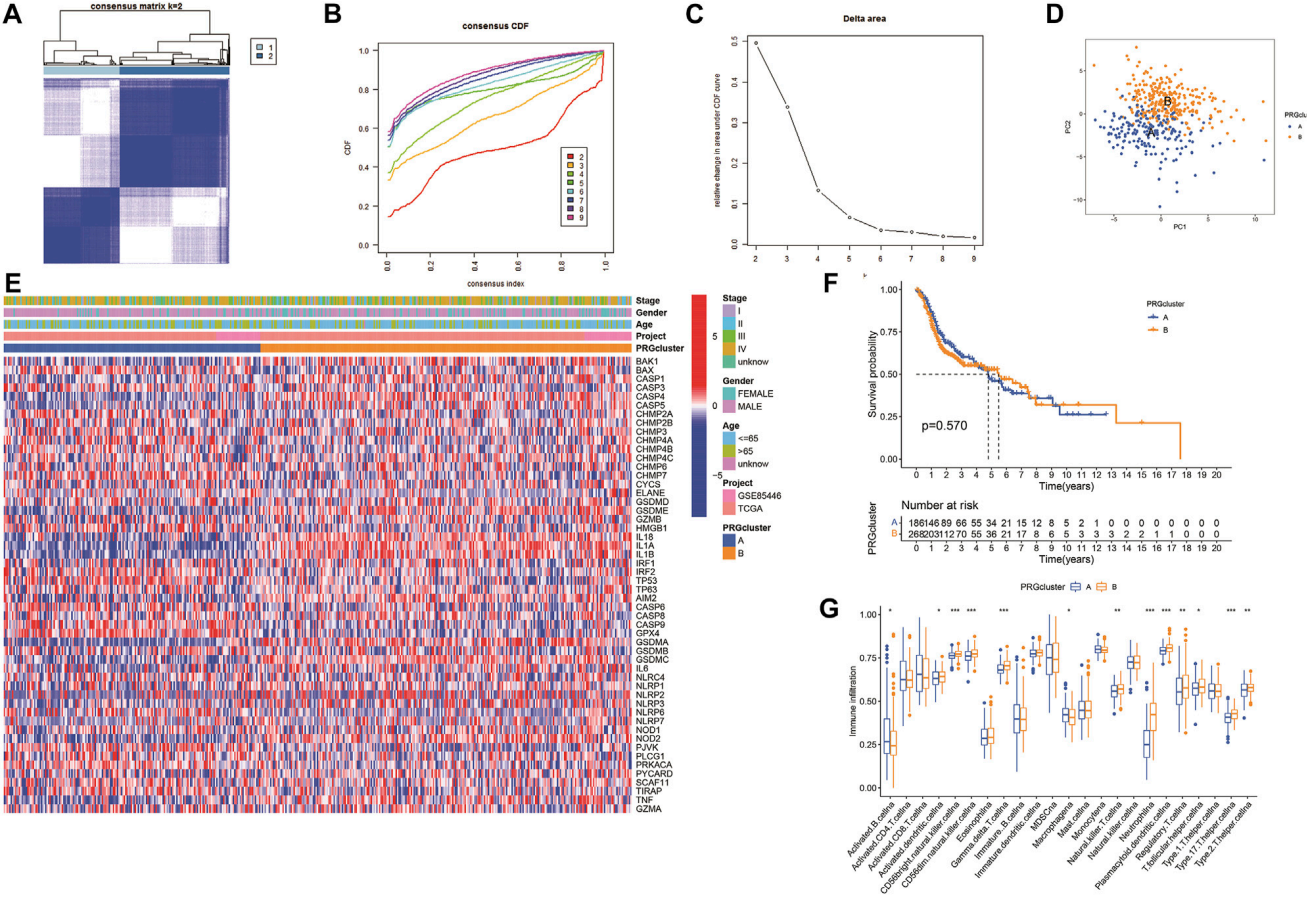
Identification of potential pyroptosis clusters in HNSCC. **(A)** Sample distribution between clusters. **(B)** Consensus clustering cumulative distribution function (CDF). **(C)** The relative variation of the area under CDF curve. **(D)** PC of TCGA according to PRG expression profiles. **(E)** The heatmap showing gene expression and clinical correlation between HNSCC clusters. **(F)** Kaplan-Meier analysis of different clusters of HNSCC patients. **(G)** Differential expression of immunocytes in HNSCC pyroptosis groups.

The expression of 52 PRGs in patients was clustered into two clusters using ConsensusClusterPlus package (Figures 4A–C), then principal component analysis (PCA) was performed to reveal the gene expression profile distribution between the two subtypes (Figure 4D). Furthermore, a heatmap showed PRG expression as well as clinical data, including age, project, gender, and survival status in clusters A and B. There was no significant difference in PRG expression between the clusters (Figure 4E), and there was no essential statistical diversity between clusters (Figure 4F). Besides, to investigate the immune spectrum of the pyroptosis clusters, we studied the location of 23 immune cells in each cluster, showing that the penetration of natural immune cells, including 13 immune cells, increased significantly (Figure 4G).

### GO enrichment and KEGG pathway analyses

**FIGURE 5:**
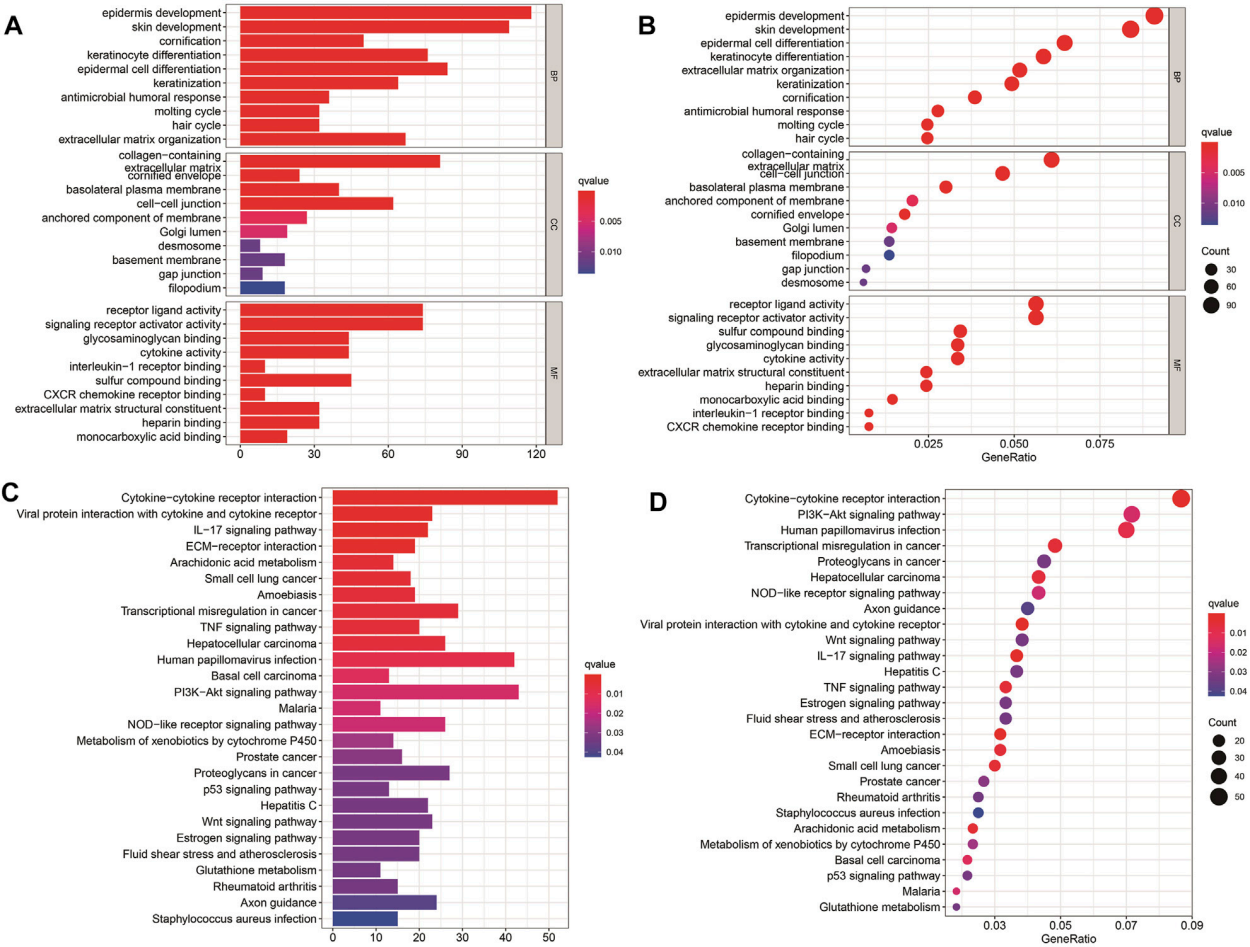
Results of GO and KEGG analyses. **(A–D)** GO and KEGG pathway analyses of DEGs between high- and low-risk groups.

To further explore the potential biological processes and pathways that lead to the molecular heterogeneity between high- and low-risk groups, diversity analysis was conducted to confirm DEGs involved in OS risk features. GO enrichment analysis suggested that DEGs were principally involved in skin development, *epidermis* cell differentiation, *epidermis* development, and collagen-containing (Figures 5A, B). KEGG pathway analysis disclosed that DEGs were mainly enriched in cytokine-cytokine receptor interaction, human papillomavirus infection, and Pl3K-Akt signaling pathway (Figures 5C, D).

### Survival analysis and variance analysis in pyroptosis-related genes correlated with prognosis

**FIGURE 6:**
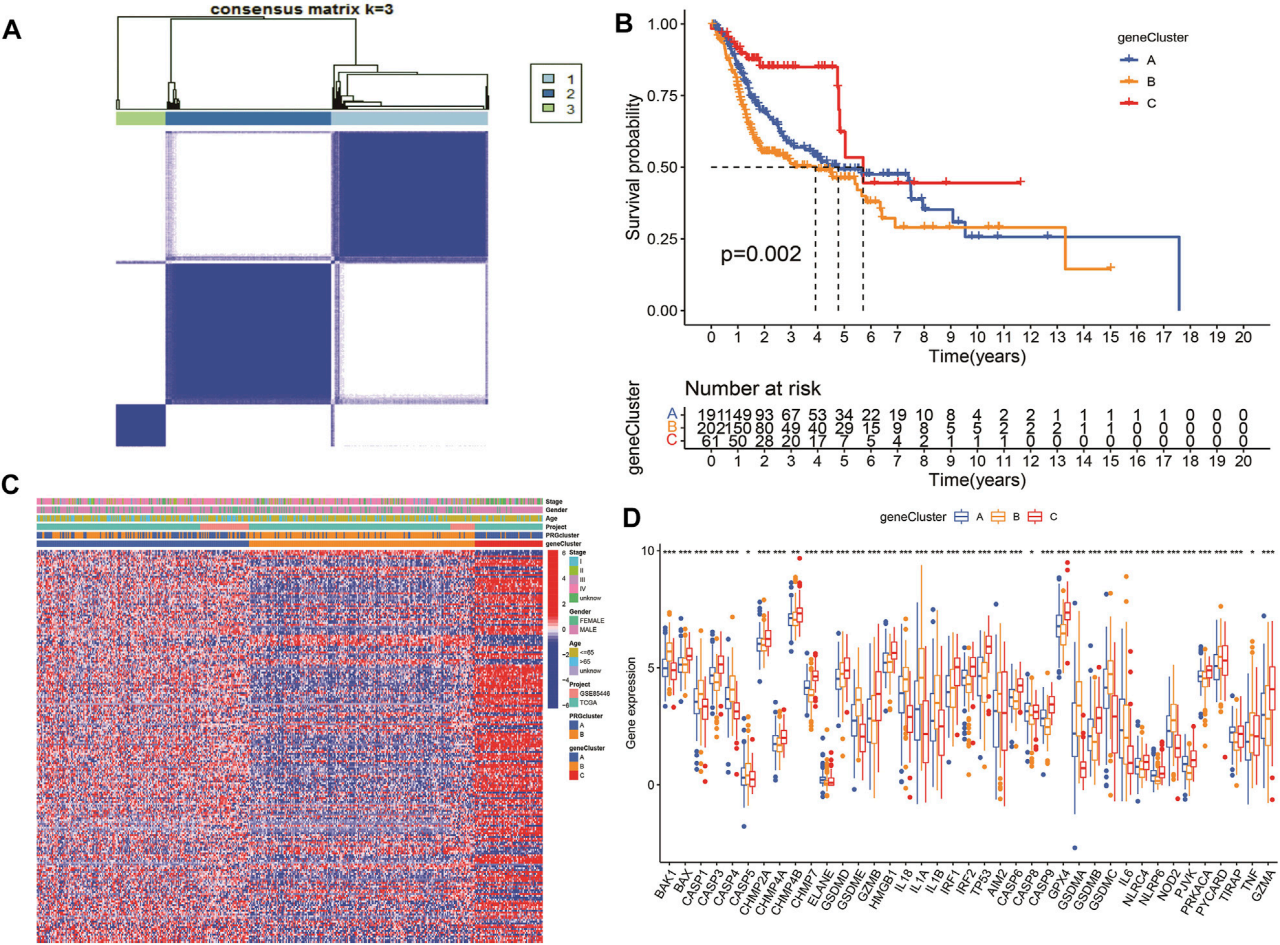
Survival analysis and variance analysis in pyroptosis-related genes correlated with prognosis. **(A)** Gene sample distribution of different clusters. **(B)** Different gene clusters in survival probability. **(C)** Gene expression and clinical correlation in different clusters showed in the heatmap clearly. **(D)** Differential expression of PRRs between the three groups. Asterisk represents the statistical *p*-value (*p < 0.05; **p < 0.01; ***p < 0.001).

**FIGURE 7:**
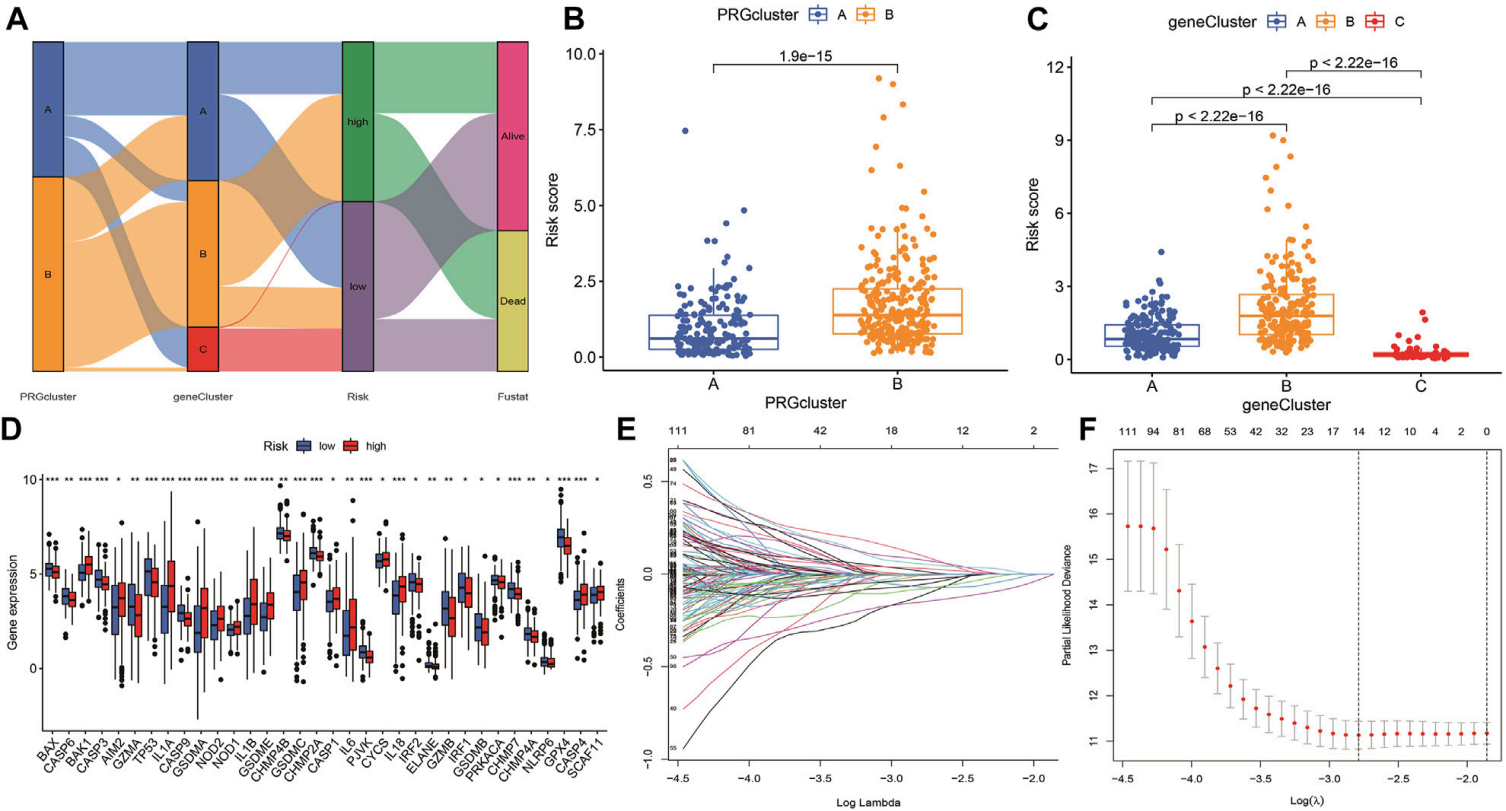
Alluvial diagram showing the changes of risk, clusters, and fustat. **(A)** The changes in clusters, risk, and fustat shown in Alluvial diagram. **(B)** The risk scores of pyroptosis were compared between the two PRG groups. Blue box representation cluster A, orange box representation cluster B. **(C)** The risk scores of pyroptosis in three gene clusters were compared. The blue box represents cluster A, the orange box represents cluster B, and the green box represents cluster C. **(D)** LASSO regression in 34 PRGs. **(E)** LASSO regression of prognostic genes **(F)** cross-validation of LASSO regression.

According to the selected prognostic PRGs, patients were clustered into three clusters (Figure 6A), with a statistical difference between gene clusters in survival probability (*p* = 0.002) (Figure 6B). Furthermore, the heatmap showed the expression of PRGs and varied clinical data, as well as the survival rate, age, grade, and TNM in clusters A–C, withPRGs being higher in group C (Figure 6C). Considering that PRGs play a vital role in the immune response regulation, PRG expression was compared between different groups (Figure 6D). The distribution of Alluvial plots in different PRG clusters, gene clusters, treatment, and PRG-related clusters is displayed in Figures 7A–C. Univariate Cox regression analysis was performed on 34 DEGs to select the survival-related genes (Figure 7D). Nine genes (TTLL1, TRIML2, DYNC1I1, KLHL35, CAMK2N1, TNFRSF18, GLDC, SPINK5, and DKK1) were further analyzed using LASSO Cox regression (Figures 7E, F).

### Construction and validation of a predictive nomogram

**FIGURE 8:**
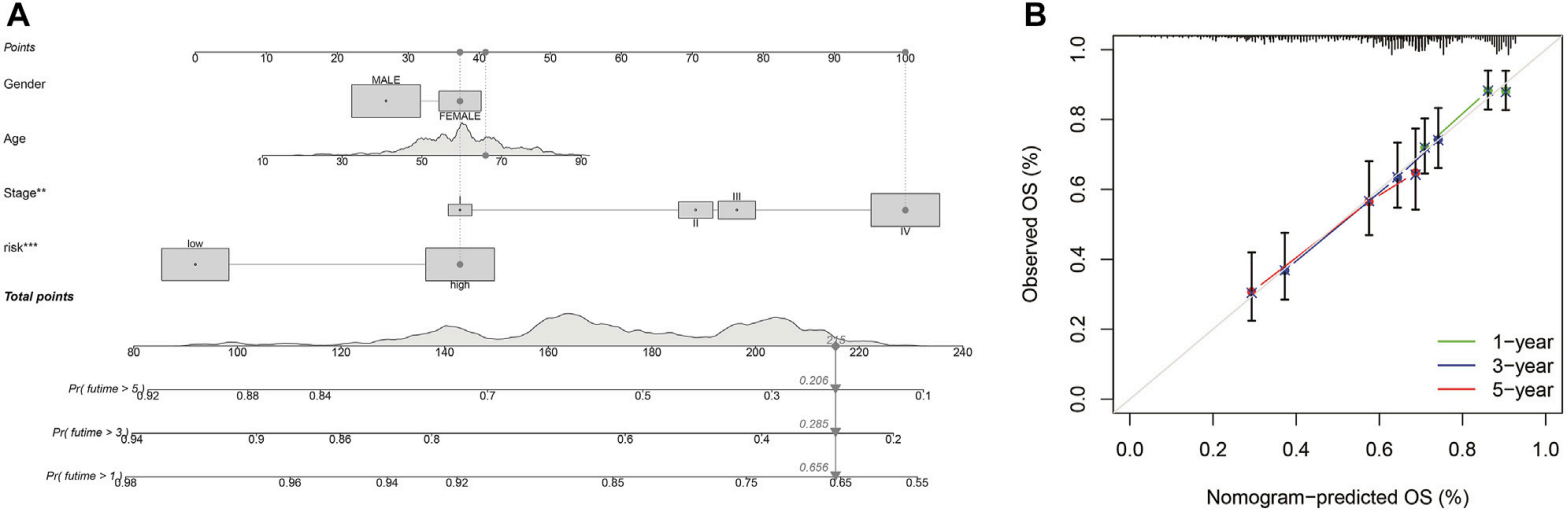
Nomogram and calibration **(A)** Nomogram to predict the 1-, 3-, and 5-years overall survival rates of HNSCC patients. **(B)** The calibration plots of the nomogram.

Different molecular HNSCC subtypes are characterized by distinctive gene expression profiles and have different features in tumor progress, therapy, and prognosis. Therefore, based on PRGs in these three subtypes, we selected nine genes (TTLL1, TRIML2, DYNC1I1, KLHL35, CAMK2N1, TNFRSF18, GLDC, SPINK5, and DKK1) related to HNSCC prognosis and conducted a new prognostic feature for HNSCC using LASSO regression algorithm program and univariate Cox regression analysis. HNSCC patients were divided into low-risk and high-risk groups based on their median risk score. The risk status, age, gender, and stage were considered the final parameters in the nomogram (Figure 8A). Based on the calibration curve of OS nomograms, OS prediction conformed to the ascertained OS (Figure 8B). Overall, the above results confirm that the nomogram has a good predictive ability to predict the survival time of HNSCC patients.

**FIGURE 9:**
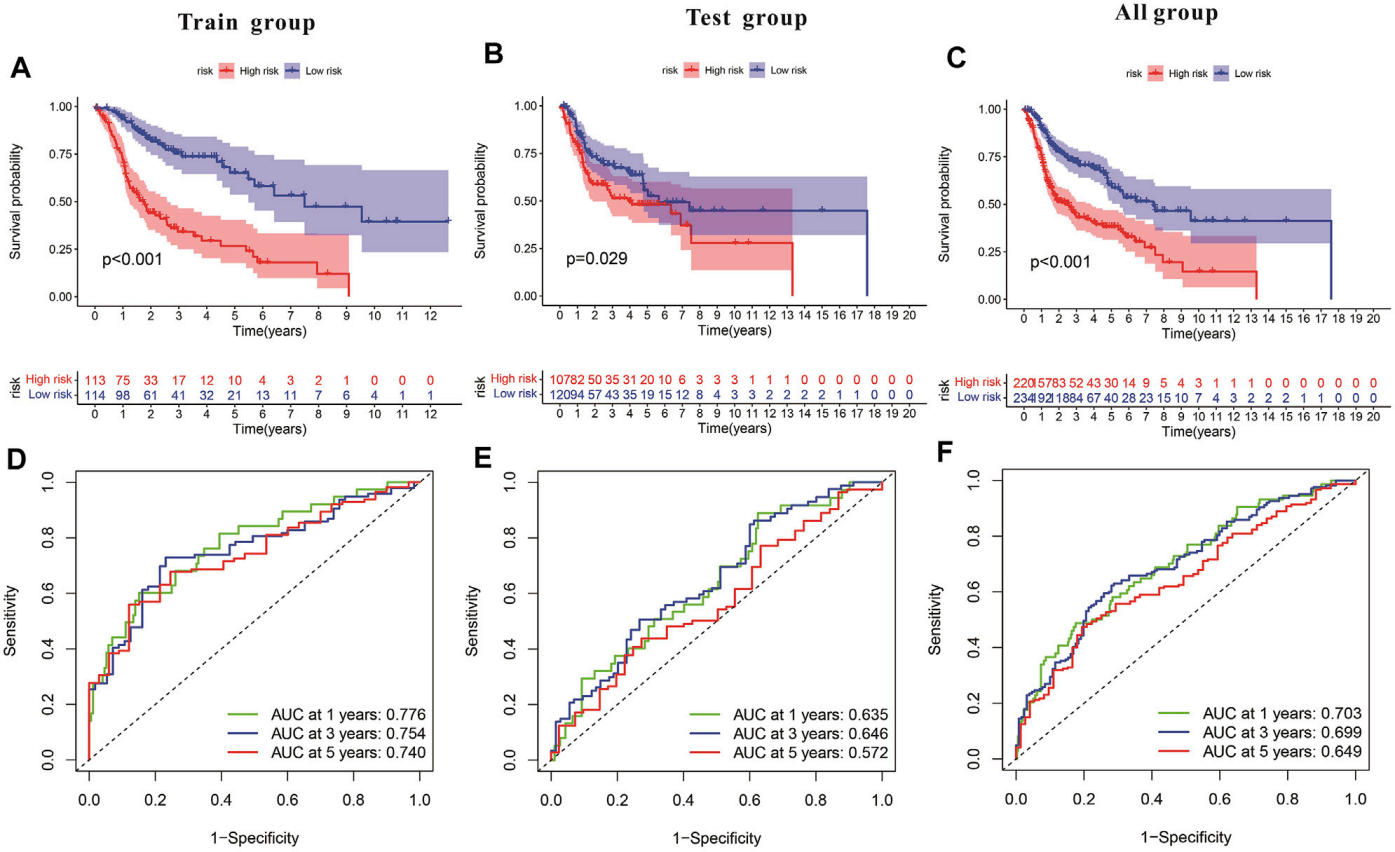
Characteristics of the pyroptosis-related gene signature in training and testing cohorts. **(A–C)** Kaplan-Meier analysis of training and testing cohorts and all groups revealed that patients at high risk had poorer survival. **(D–F)** Risk model in ROC curves at 1, 3, and 5 years.

**FIGURE 10:**
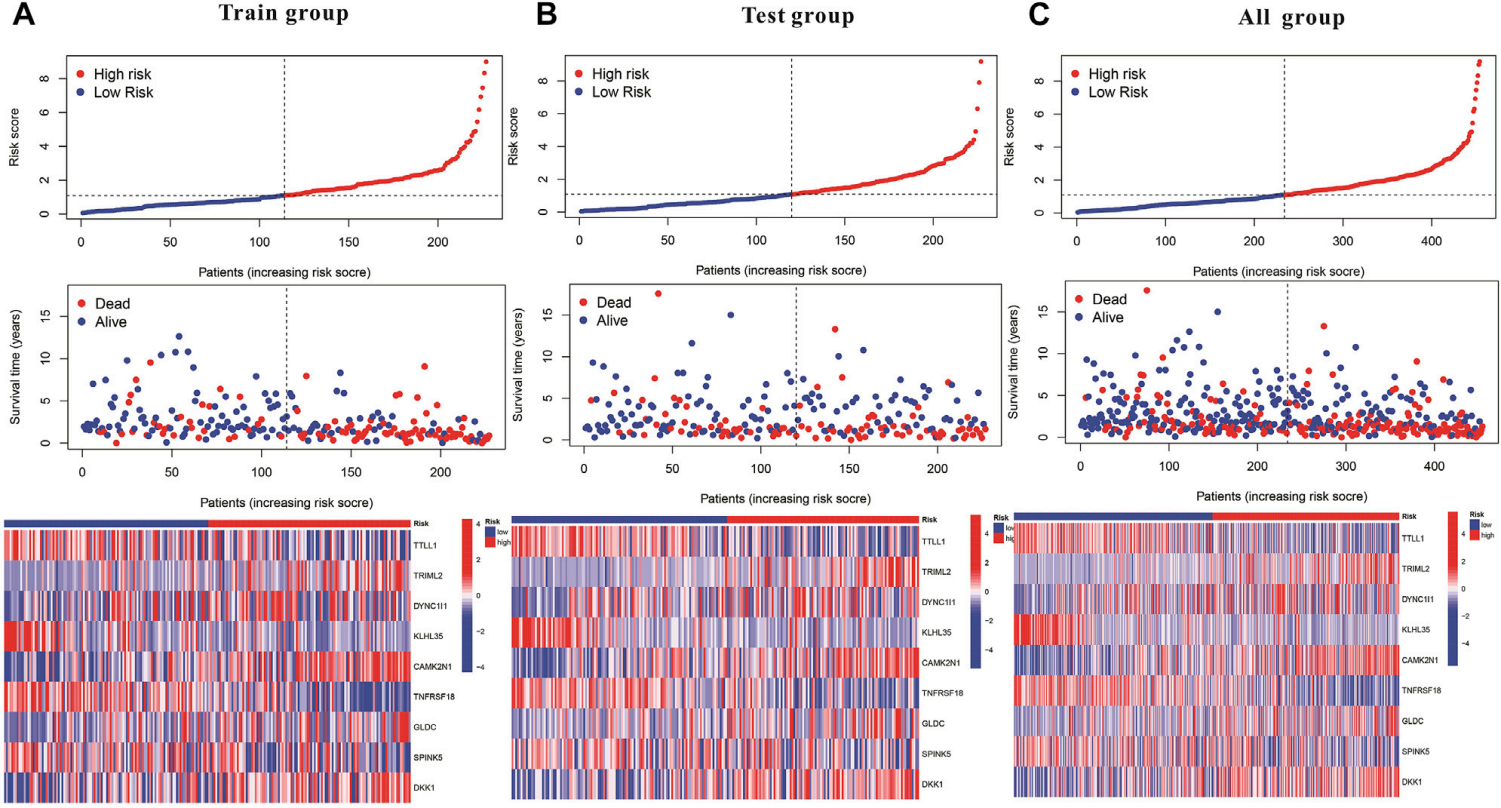
Kaplan–Meier and prognostic risk curves. **(A)** Train group. **(B)** Test group. **(C)** All groups. The dotted line indicates the personal inflection point of the risk score, and patients are divided into low- (green) and high-risk (red) groups. Red dots represent patients who are dead, and green dots represent those who are alive. As the risk score increased, numerous patients died. Colors from blue to red indicate low to high expression levels.

Subsequently, a Kaplan-Meier analysis was performed of OS between the high-risk and low-risk teams with the training cohort, testing cohort, and all groups (Figures 9A–C). The area underneath ROC curve (AUCs) for survival times of 1, 3, and 5 years indicated that this gene signature performs well in predicting HNSCC survival (Figures 9D–F). With the increase in risk score, the risk of death increases, and the survival time reduces. The risk heatmap indicates that TRIML2, CAMK2N1, and DKK1 were upregulated in the high-risk cluster, suggesting their tumor-promoting role (Figures 10A–C).

### Expression of pyroptosis-related genes in tissues by qRTPCR

**FIGURE 11:**
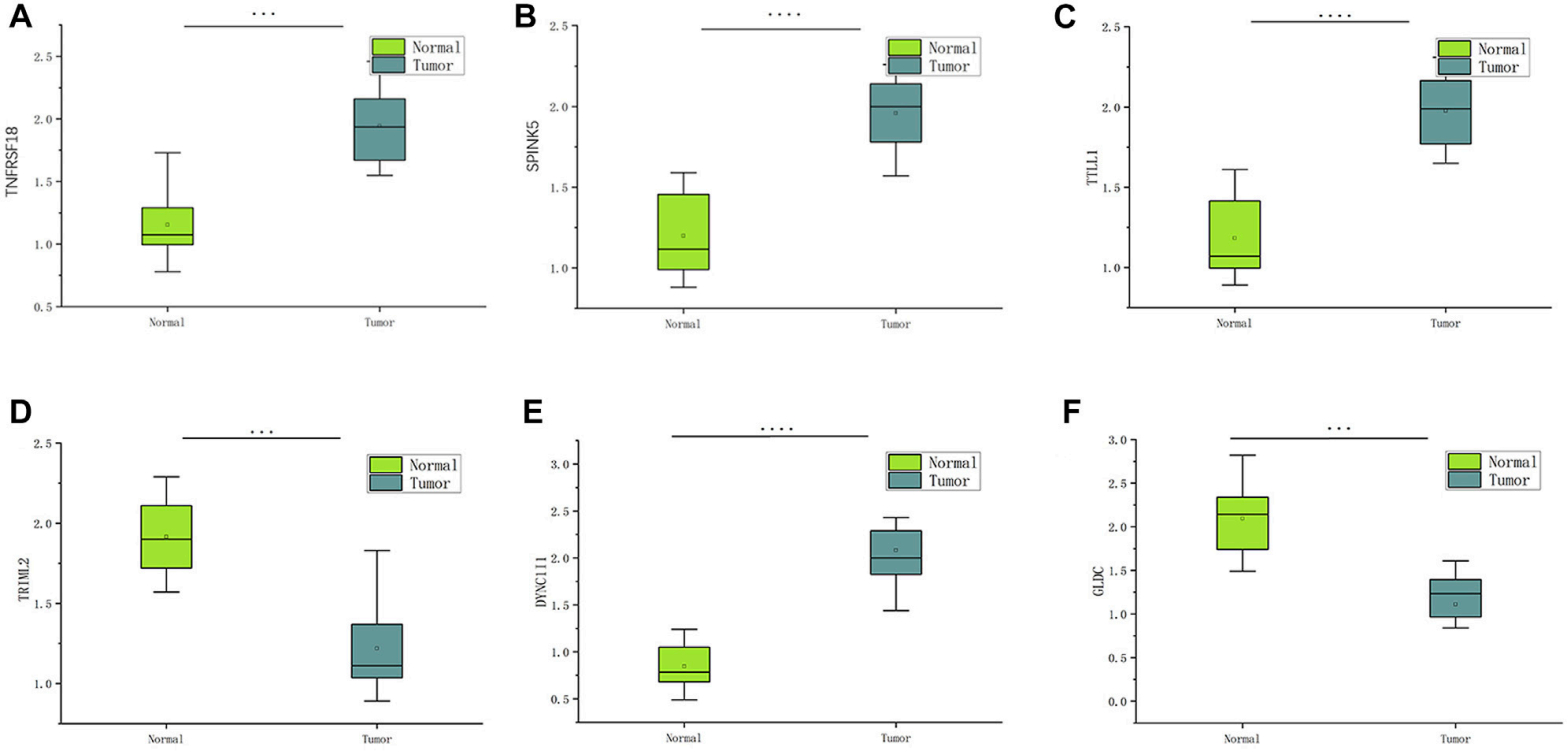
The expression of six pyroptosis-related genes in HNSCC and paracancerous tissues was measured by qRT-PCR. ∗∗∗*p* < 0:001and ∗∗∗∗*p* < 0:0001.

The information from database revealed that pyroptosis-related genes related to HNSCC prognosis. To verify the feasibility of the prognostic model, we measured the expression of genes in HNSCC tissues and paired normal paracancerous tissues by qRT-PCR. The primers utilized are presented in Table 1. Based on human paired HNSCC tissues obtained by surgery, we validated the differential expression of six risk genes included in the risk model by qRT-PCR assays. The differential analysis revealed that TRIML2, DYNC1I1, TRIML2, and TTLL1 was significantly upregulated in HNSCC tissues, while GLDC and TNFRSF18 were downregulated in HNSCC tissues (Figure 11). We validated the reliability of pyroptosis-related genes as prognostic markers for HNSCC using qRT-PCR experiments.

**TABLE. 1:** Primer information for qRT-PCR.

Primer	Sequence	Primer length	Product size
H-TTLL1-S	CTG​CTT​TGA​ATG​CTA​TGG​CTA​CG	23	207 bp
H-TTLL1-A	ACT​TCC​TTA​GGT​GGC​GAC​TTG​TT	23	
H-TRIML2-S	CAG​TCC​CAG​GAG​CAC​AAA​CAT	21	158 bp
H-TRIML2-A	TCA​TCG​CCA​TTC​TTT​CTT​GCT​C	22	
H-DYNC1I1-S	CTT​GGT​GGT​TGG​TGG​GAC​TTA​CT	23	161 bp
H-DYNC1I1-A	TGA​GGT​TAT​GAG​CAT​TCT​GGG​TC	23	
H-KLHL35-S	CCT​TCT​CAC​AGC​GGT​GTC​TC	20	128 bp
H-KLHL35-A	ACA​CAC​AGT​GAC​TCC​ACA​GC	20	
H-TNFRSF18-S	ATG​TGT​GTC​CAG​CCT​GAA​TTC​C	22	271 bp
H-TNFRSF18-A	CAG​TCG​ATA​CAC​TGG​AAG​CCA​AA	23	
H-DKK1(1)-S	CAG​GCG​TGC​AAA​TCT​GTC​TC	20	139 bp
H-DKK1(1)-A	GCA​CAG​TCT​GAT​GAC​CGG​AG	20	
H-CAMK2N1-S	GCA​GGA​CAC​CAA​CAA​CTT​CTT​C	22	151 bp
H-CAMK2N1-A	AGG​TGC​CTT​GTC​GGT​CAT​ATT​TT	23	
H-GLDC-S	GGT​CTG​ATG​TCT​CGC​ACC​TAA​A	22	170 bp
H-GLDC-A	GCA​TCC​TCA​TTC​CGC​TTT​AGT​G	22	
H-SPINK5-S	ATC​AAA​TGG​GAC​TGG​ATC​AGA​AT	23	138 bp
H-SPINK5-A	CTT​CCC​TTT​CCA​GTT​TTT​CCT​TAC	24	
R-GAPDH-S	CTG​GAG​AAA​CCT​GCC​AAG​TAT​G	22	
R-GAPDH-A	GGT​GGA​AGA​ATG​GGA​GTT​GCT	21	

### Immune characterization analysis and immune checkpoint prediction

**FIGURE 12:**
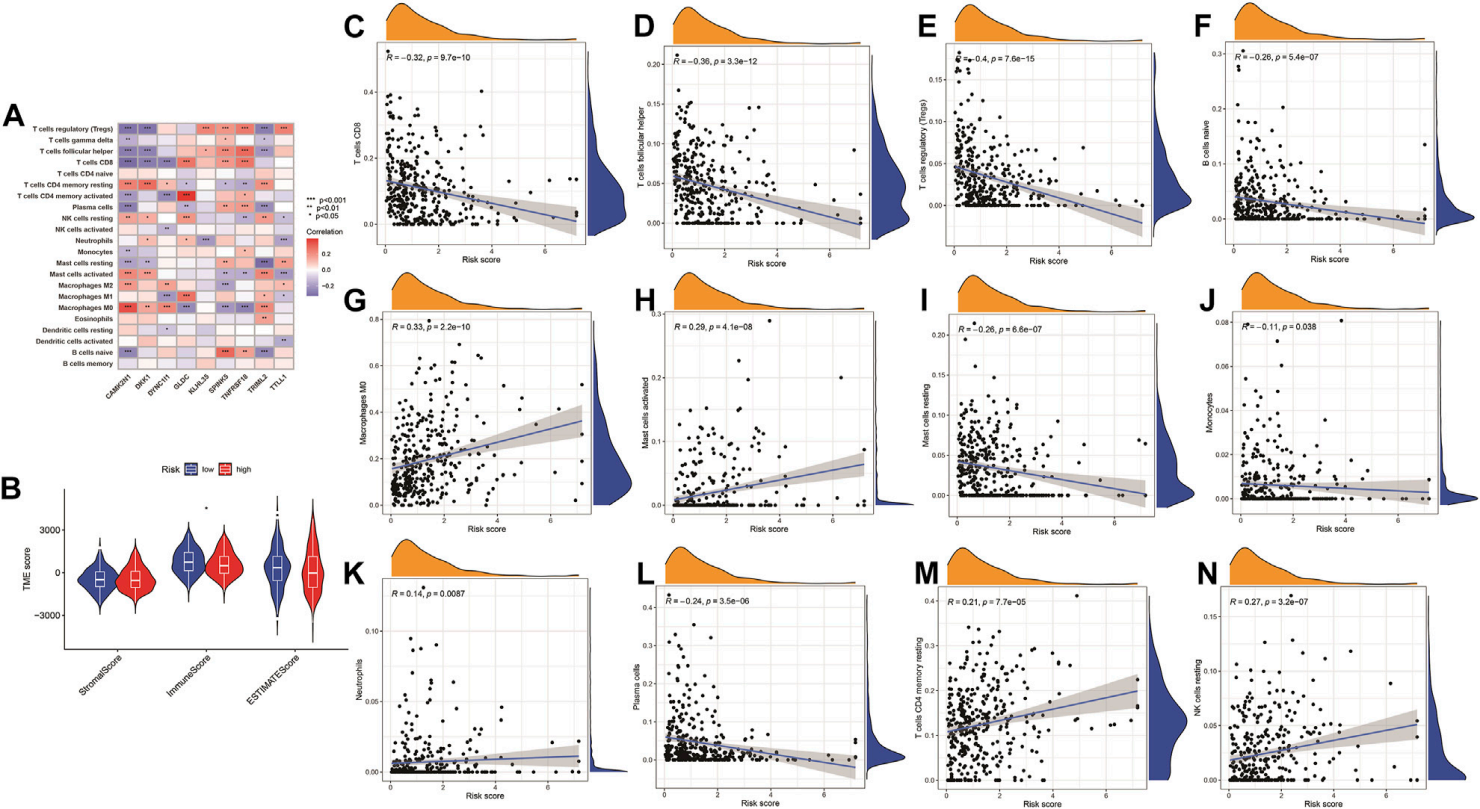
Immune checkpoint analysis. **(A)** The expression of PRGs and immune cells between low- and high-risk groups. *p < 0.05, **p < 0.01; ***p < 0.001. **(B)** Violin plot for a stromal score, immune score, ESTIMATE score, and tumor purity score between high- and low-risk groups. **(C–N)** Correlation analysis of the risk model and immune cells infiltration.

Pearson correlation analysis was conducted to analyze the correlation between the nine genes (TTLL1, TRIML2, DYNC1I1, KLHL35, CAMK2N1, TNFRSF18, GLDC, SPINK5, and DKK1) associated with pyroptosis and twelve immune cell types (B cells naïve, M0 macrophages, resting mast cells, activated mast cells, monocytes, resting NK cells, neutrophils, plasma cells, CD8 T-cells, CD4 memory resting T-cells, follicular helper T-cells, and regulatory T-cells (Tregs). The comparison of high-risk and low-risk groups of the stromal, immune, ESTIMATE, and tumor purity scores in the Violin Plot indicated that only the immunescore was statistically significant (Figure 12).

### Correlation analysis of immune cells in different software and differences in drug sensitivity between high-risk and low-risk groups

**FIGURE 13:**
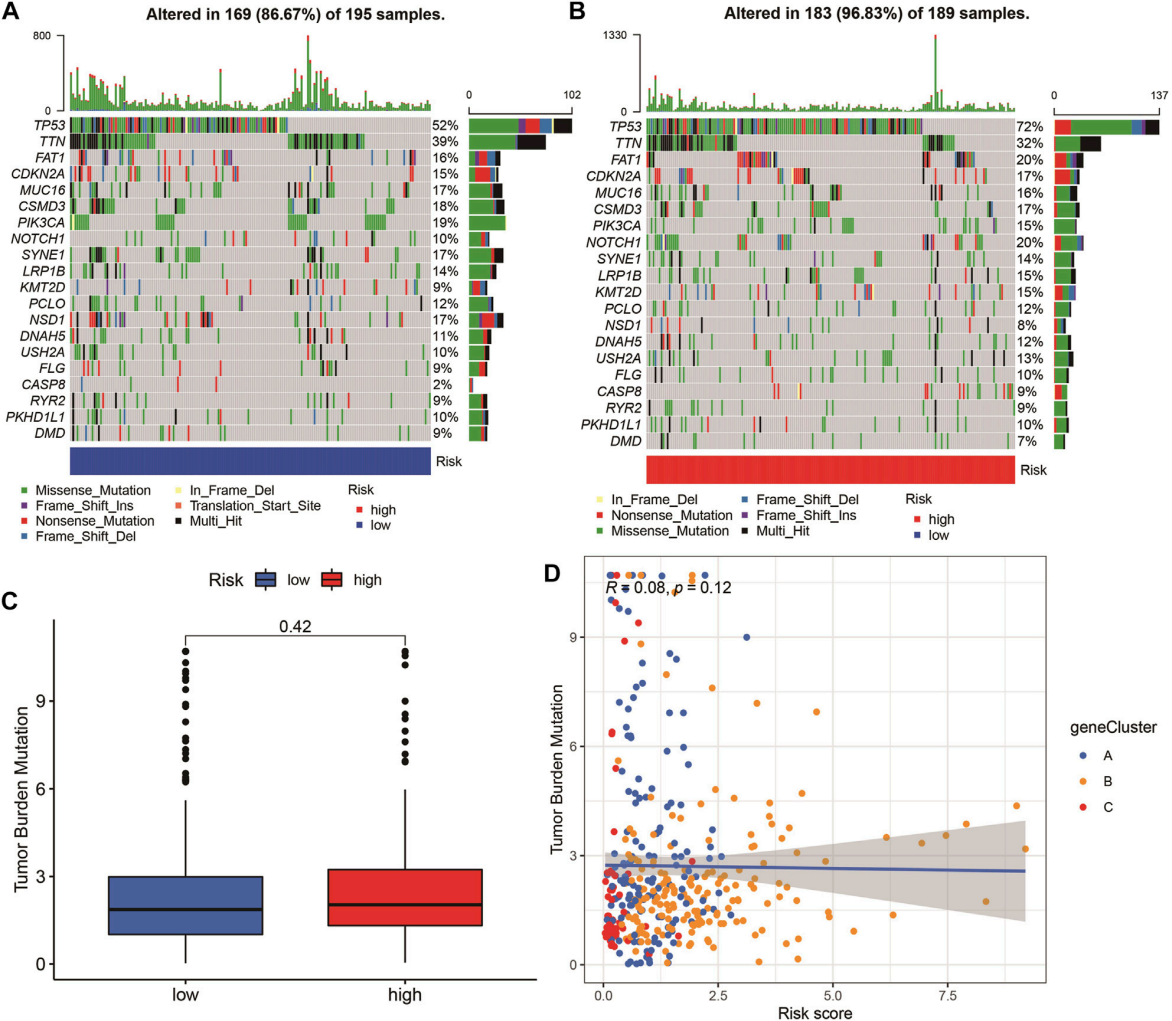
Tumor mutation burden (TMB) analyses. **(A–B)** The top 20 driver genes with the highest mutation frequencies in high and low pyroptosis score groups **(C)** Boxplot illustrates that TBM value in the high-risk group was not significantly higher (*p* = 0:42). **(D)** Scatter plots of correlation analysis of risk score and TBM value.

**FIGURE 14:**
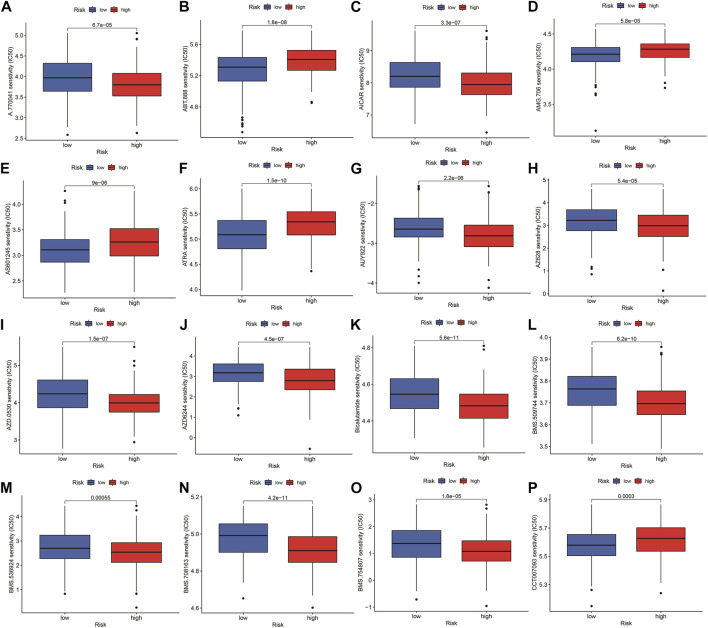
Comparison of drug sensitivity between high- and low-risk groups. **(A–P)** Drug sensitivity between high- and low-risk groups.

The highest 20 driver genes with the best mutation frequency of TP53 and TTN were considerably different between high (Figure 13A) and low-risk groups (Figure 13B). As the risk score increased, TMB value also increased but did not reach significance (*p* = 0:047) (Figure 13C). However, the relationship between TMB and risk score was insignificant (R = 0:08, *p* = 0:12) (Figure 13D). To determine if the risk score and chemotherapeutic efficacy are linked in treating HNSCC, we identified the relationship between risk scores and the sensitivity to sixty chemotherapeutic drugs. There was discernible variation in drug sensitivity among most types of chemotherapeutic medicines between the two groups (Figure 14A–P).

## Discussion

The incidence and mortality of HNSCC have recently increased, and HNSCC has become the foremost fatal malignancy in adults (Ferlay et al., 2019). However, traditional histopathological characteristics (stage, grade, and tumor size) might not meet the requirements of diagnoses and prognoses (Gatta et al., 2015; Sacco and Cohen, 2015; Ferlay et al., 2019). Therefore, it is necessary to explore new HNSCC biomarkers to fulfill the clinical necessities for the identification and prognosis of HNSCC. As lytic programmed death, pyroptosis is a crucial mechanism for HNSCC pathological development and has been widely researched in tumor models (Kist, 2021; Yu et al., 2021). Recent studies have revealed that pyroptosis is a new procedural cell death that plays an important role in tumor growth and therapy mechanisms (Kovacs and Miao, 2017), such as head and neck tumors (Wellenstein and de Visser, 2018; Cai et al., 2021; Yuan et al., 2021). Prognostic markers related to pyroptosis have been constructed for gastric and ovarian tumors with a good prognostic potential (Shao et al., 2021; Ye et al., 2021). However, the interaction of PRGs in HNSCC, as well as the potential ability to predict the prognosis of HNSCC patients and comprehensive analysis of PRG for prognosis prediction and targeted treatment in HNSCC patients, remain unclear.

The present study investigated the potential prognostic significance of PRGs and investigated the expression of PRGs in 413 HNSCC patients. Such patients were classified as high or low-risk based on their median risk score, with the risk model considerably differentiating the clinical features of low and high-risk HNSCC patients; hence, it was a good independent prognostic indicator. Notably, GO, KEGG, GSEA, and ssGSEA analyses revealed the varied immune status of the high and low-risk groups. Nine genes associated with HNSCC prognosis (TTLL1, TRIML2, DYNC1I1, KLHL35, CAMK2N1, TNFRSF18, GLDC, SPINK5, and DKK1) were used to construct a new prognostic signature for HNSCC by LASSO regression algorithm and univariate Cox regression.

Based on human paired HNSCC tissues obtained by surgery, we validated the differential expression of six risk genes included in the risk model by qRT-PCR assays. The differential analysis revealed that DYNC1I1, SPINK5, TNFRSF18, and TTLL1 was significantly upregulated in HNSCC tissues, while GLDC and TRIML2 were downregulated in HNSCC tissues. One research shows that DYNC1I1 gene expression was up-regulated and further led to activation of the AKT/ERK signaling pathway to promote hepatocellular carcinoma (HCC) progression (Liu et al., 2022). Daniel N Frank found that PINK5 variants confer susceptibility to non-syndromic Otitis media (OM). These variants potentially contribute to middle ear pathology through alteration of head and neck microbiota and facilitation of entry of opportunistic pathogens into the middle ear (Frank et al., 2021). P Vogel investigating the role of TTLL1 and polyglutamylation of tubulin in cilia and flagella should advance an understanding of the biogenesis and function of these organelles in mammals and have potential diagnostic and therapeutic applications (Vogel et al., 2010). Previous studies have shown TRIML2 knockdown oral squamous cell carcinoma (OSCC) cells showed decreased cellular proliferation by cell-cycle arrest at G1 phase and TRIML2 might play a significant role in tumoral growth (Hayashi et al., 2019). Detailed mechanisms need to be explored further. In summary, this gene signature was highly effective and a new means for predicting HNSCC prognosis.

Age, stage, and risk score were considerably related to OS by Cox regression analysis. Actually, a lot of predictive models are widely established and reported in many studies by univariate Cox regression and Lasso Cox regression analysis. Cao et al. (2017) identify a lncRNA prognostic signature model using the orthogonal partial least squares discrimination analysis (OPLS-DA) and 1.5-fold expression change criterion methods. (OPLS-DA) are powerful statistical modeling tools that provide insights into separations between experimental groups based on high-dimensional spectral measurements from NMR, MS or other analytical instrumentation (Blasco et al., 2015). LASSO Cox regression analysis is a method for variable selection and shrinkage in Cox proportional hazards model that constructs a penalty function to obtain a more refined mode. The lasso is a popular selection method in Cox regression, but its results depend heavily on the penalty parameter *λ* (Ternès et al., 2016).

Furthermore, high-risk patients determined based on this feature were confirmed to have a higher TMB. The TMB has become an important factor in disease progression and clinical relapse in HNSCC patients. A better understanding of the HNSCC biology, especially the interaction between cancer cells and their surrounding TME, may help identify new biomarkers, enabling patient stratification for clinical decision-making (Van den Bossche et al., 2022). Also, targeting PRGs may be a promising strategy for treating HNSCC. The invasive immune cell is a crucial component of TME (Wellenstein and de Visser, 2018), and most immune cells were positively correlated with the risk score, with variations in the composition of immune cell types between risk groups. Many studies have revealed that in the immune response, the state of the gene adjusts the function of immune cells (Chen et al., 2018; Shao et al., 2021). This risk model based on PRGs may be promising for the clinical prediction of prognoses and immunotherapeutic responses in HNSCC patients.

The combination of immunotherapy, chemotherapy, radiotherapy, and targeted medical help inhibit tumor progression through synergistic mechanisms and improve poor prognosis in cancer patients. Although tumor development is complex, the inflammatory response may be a relevant factor. Pyroptosis is a new type of cellular necrosis, and its characteristics are the release of many inflammatory factors. Chronic inflammatory reactions can result in native tissue damage and neoplastic lesions.

This study has many advantages. First, the prognostic signature accurately predicts the OS for HNSCC patients. Additionally, it is considerably related to TMB and immune infiltration, indicating its biomarker potential in HNSCC. Non-etheless, this study has some limitations. First, all information for the analysis was retrieved from online databases, implying that future *in vivo* and *in vitro* research is required to verify these results. Second, post-translational modifications play an important role in signal transduction and cellular function, but the translational modifications of these genes were not explored.

## Conclusion

A nine pyroptosis-related gene signature was constructed to better predict the prognosis and immune status of patients with head and neck squamous cell carcinoma.

## Data Availability

The original contributions presented in the study are included in the article/supplementary material, further inquiries can be directed to the corresponding author.
